# Há uma Função para o Telemonitoramento na Insuficiência Cardíaca?

**DOI:** 10.36660/abc.20220034

**Published:** 2022-03-10

**Authors:** Mônica Samuel Avila, Deborah de Sá Pereira Belfort

**Affiliations:** 1 Hospital das Clínicas Faculdade de Medicina Universidade de São Paulo São Paulo SP Brasil Instituto do Coração do Hospital das Clínicas da Faculdade de Medicina da Universidade de São Paulo, São Paulo, SP – Brasil

**Keywords:** Insuficiência Cardíaca/fisiopatologia, Telemonitoramento, Hospitalização, Serviços de Emergência

A insuficiência cardíaca (IC) é a principal causa de hospitalização cardiovascular no mundo. Os índices de mortalidade variam de 5 a 15%, e até 50% dos pacientes são readmitidos na emergência no período de 90 dias após a alta.^1^ Várias estratégias foram implementadas nos últimos anos para evitar a readmissão hospitalar, e a telemedicina é um campo que está crescendo nesse cenário. O uso de tecnologias de telecomunicação traz vantagens possíveis em relação à assistência presencial, superando barreiras organizacionais e geográficas. Entretanto, resultados divergentes em ensaios randomizados avaliando a eficácia da telemedicina na redução de hospitalizações e mortalidade por insuficiência cardíaca^2^ desencorajaram o uso rotineiro de recursos digitais na prática clínica até a pandemia da COVID-19.

Nesta edição dos Arquivos Brasileiros de Cardiologia, um estudo observacional retrospectivo avaliou o impacto de um programa avançado de telemonitoramento em uma população portadora de insuficiência cardíaca.^3^ Foram incluídos trinta e nove pacientes, e os pesquisadores compararam o número de hospitalizações um ano antes do programa ao número de hospitalizações durante o programa. O programa usou sinais vitais e variáveis, tais como, frequência cardíaca, pressão arterial, variação de peso, oxigenação do sangue periférico, temperatura, e um eletrocardiograma inicial com 3 eletrodos. A análise final incluiu trinta e quatro pacientes. Os autores relataram uma redução de 66% nas admissões de emergência, e uma redução de 68% nas hospitalizações por insuficiência cardíaca, considerando os próprios pacientes como controle.

O pequeno número de participantes, a natureza observacional retrospectiva do estudo e a ausência de participantes de controle simultâneos fazem com que esses resultados sejam apenas geradores de hipótese; entretanto, eles estão em consonância com a literatura atual. Embora ensaios controlados randomizados (ECT) na última década tenham demonstrado resultados divergentes em relação à eficácia da telemedicina na insuficiência cardíaca,^2^ revisões sistemáticas demonstraram redução de hospitalizações e mortalidade entre essa população. Uma revisão sistemática Cochrane de 2015, que incluiu apenas ECT, avaliou o uso de suporte telefônico estruturado ou de telemonitoramento doméstico não invasivo comparado à prática padrão para pessoas com insuficiência cardíaca.^4^ Ela demonstrou que o telemonitoramento não invasivo reduziu a mortalidade global (TR 0,80, IC 95% 0,68 a 0,94) e hospitalizações relacionadas à insuficiência cardíaca (TR 0,71, IC 95% 0,60 a 0,83). Outra revisão sistemática que incluiu apenas ECT e 11.450 pacientes, publicada em 2020, confirmou resultados semelhantes.^5^

As diretrizes atuais também são divergentes quanto à classe de recomendação sobre telemedicina com pacientes portadores de insuficiência cardíaca. A Diretriz da Sociedade Brasileira de Cardiologia sobre Telemedicina na Cardiologia orienta os cardiologistas a usarem estratégias de telemonitoramento com suporte telefônico estruturado na insuficiência cardíaca para reduzir as hospitalizações (recomendação classe IA) e mortalidade (recomendação classe IIA),^6^ e isso está alinhado à Atualização de Tópicos Emergentes da Diretriz Brasileira de Insuficiência Cardíaca - 2021 (recomendação classe IIA para mortalidade e hospitalizações).^7^ A diretriz de insuficiência cardíaca da Sociedade Europeia de Cardiologia, entretanto, não faz nenhuma recomendação sobre monitoramento remoto não invasivo.^8^ enquanto a Sociedade Americana de Cardiologia recomenda sistemas eficazes para coordenar o tratamento da IC para oferecer a terapia médica recomendada pelas diretrizes e evitar hospitalizações (recomendação classe I)).^9^

Esse estudo retrospectivo não esclarece dúvidas sobre a eficácia da telemedicina na insuficiência cardíaca; entretanto, ele chama a atenção para um tópico relevante não apenas na insuficiência cardíaca, mas em todas as áreas clínicas. A avaliação presencial se tornou limitada no sistema de saúde depois do novo coronavírus, levando à necessidade crescente de formas alternativas de avaliação clínica.^10^ A pandemia da COVID-19 impulsionou o desenvolvimento de ferramentas de monitoramento remoto, e novos ensaios precisam ser desenhados para analisar o papel da telemedicina após essas mudanças globais, e para incentivar o uso rotineiro dessa ferramenta na prática clínica.
