# Supplementary material for: Há uma Função para o Telemonitoramento na Insuficiência Cardíaca?
Source: Arq Bras Cardiol. 2022 Mar 10;118(3):605–6. [Article in Portuguese] doi: 10.36660/abc.20220034 (PMC8959035; doi:10.36660/abc.20220034)
Supplement: Translation

# Translation: Is There a Role for Telemonitoring in Heart Failure?

**Authors:** Mônica Samuel Avila, Deborah de Sá Pereira Belfort

**Affiliations:** 1 Brazil Instituto do Coração do Hospital das Clínicas da Faculdade de Medicina da Universidade de São Paulo, São Paulo, SP – Brazil

**Keywords:** Heart Failure/physiopathology, Telemonitoring, Hospitalization, Emergency Services

Heart failure (HF) is the leading cause of cardiovascular hospitalization in the world. Mortality rate ranges from 5% to 15%, and up to 50% of patients are readmitted in the emergency department in 90 days after discharge.^1^ Different strategies have been implemented in recent years to avoid readmission, and telemedicine is a growing field in this scenario. The use of telecommunication technologies brings potential advantages when compared to in-person care, overcoming organizational and geographic barriers. However, divergent results in randomized trials evaluating the efficacy of telemedicine in reducing heart failure hospitalizations and mortality^2^ discouraged the routine use of digital resources in clinical practice until the COVID-19 pandemic.

In this issue of Arquivos Brasileiros de Cardiologia, retrospective observational research evaluated the impact of an advanced telemonitoring program in a heart failure population.^3^ Thirty-nine patients were included, and the researchers compared the number of hospitalizations one year before the program, with hospitalizations during the program. The program used vital signs and variables such as heart rate, blood pressure, weight variation, peripheral blood oxygenation, temperature, and a seminal 3-derivation electrocardiogram. Thirty-four patients were included in the final analysis. The authors reported a 66% reduction in emergency department admissions and a reduction of 68% in heart failure hospitalizations, considering the patients themselves as controls.

The small number of participants, the retrospective observational nature of the study, and the absence of simultaneous control participants make these results only hypothesis generating results, but they do run in line with current literature. Although randomized controlled trials (RCTs) in the last decade showed divergent results regarding the efficacy of telemedicine in heart failure,^2^ systematic reviews showed a reduction in hospitalizations and mortality among this population. A Cochrane systematic review in 2015, including only RCTs, evaluated the use

of structured telephone support or non-invasive home telemonitoring compared to standard practice for people with heart failure.^4^ This study showed that non-invasive telemonitoring reduced all-cause mortality (RR 0.80, 95% CI 0.68 to 0.94) and heart failure-related hospitalizations (RR 0.71, 95% CI 0.60 to 0.83). Another systematic review, also including only RCTs and 11,450 patients, published in 2020, confirmed similar results.^5^

Current guidelines also diverge in the class of recommendation on telemedicine with heart failure patients. The Guideline of the Brazilian Society of Cardiology on Telemedicine in Cardiology advise cardiologists to use noninvasive telemonitoring strategies with structured telephone support in heart failure to reduce hospitalizations (class IA recommendation) and mortality (class IIA recommendation),^6^ which is in alignment with the Emerging Topics Update of the Brazilian Heart Failure Guideline - 2021 (class IIA recommendation for mortality and hospitalizations).^7^ The European Society of Cardiology Heart Failure guideline, however, does not provide any recommendation on non-invasive remote monitoring,^8^ while the American Heart Association recommend effective systems to coordinate HF care to provide the guideline-recommended medical therapy and prevent hospitalizations (class I recommendation).^9^

This retrospective research does not clear doubts about efficacy of telemedicine in heart failure, however it draws attention to a relevant theme not only in heart failure, but in all clinical areas. In-person evaluation became limited in the healthcare system after the new coronavirus, leading to a growing need for alternative means of clinical evaluation.^10^ The COVID-19 pandemic boosted the development of remote monitoring tools, and new trials need to be designed to analyze the role of telemedicine after these global changes and to encourage the routine use of this tool in clinical practice.
